# Associations of nutrition knowledge and dietary habits with stress among university employees in Saudi Arabia: mixed-methods evidence

**DOI:** 10.3389/fnut.2026.1834114

**Published:** 2026-05-26

**Authors:** Alyaa M. Zagzoog, Fatimah Y. Almujlad, Almaha M. Alanazi

**Affiliations:** Department of Community Health, Faculty of Applied Medical Sciences, Northern Border University, Arar, Saudi Arabia

**Keywords:** dietary habits, employee, health, nutrition knowledge, university, stress, Saudi Arabia

## Abstract

**Objective:**

This study examined the relationships among dietary habits, nutrition knowledge, and stress levels among Saudi university employees, and to explore perceived challenges, barriers, and facilitators of healthy eating in the workplace.

**Design:**

A mixed-methods design was used, combining an online quantitative survey with virtual semi-structured interviews.

**Setting:**

The study was conducted across universities in Saudi Arabia, focusing on workplace environments that may influence employees’ dietary behaviors, nutrition knowledge, and stress levels.

**Participants:**

A total of 232 university employees completed validated measures of nutrition knowledge using the General Nutrition Knowledge Questionnaire, dietary habits using Arabic Food Frequency Questionnaire, and stress using Arabic Stress Overload Scale, with a subsample participating in qualitative interviews to explore workplace-related experiences and barriers to healthy eating.

**Results:**

Most participants demonstrated medium to high nutrition knowledge (86.2%); however, gaps were identified in applied dietary recommendations and nutrient-specific knowledge. Irregular eating patterns, meal skipping, and low fruit and vegetable intake were significantly associated with higher stress levels (*p* < 0.05). Higher nutrition knowledge was positively associated with healthier food choices, more regular meals, and food label reading. Qualitative findings highlighted workplace demands, sociocultural and environmental constraints, the close link between nutrition and mental health, and the importance of access, awareness, and supportive environments.

**Conclusion:**

Nutrition knowledge is associated with dietary behaviors and stress among Saudi university employees. Workplace-targeted interventions integrating nutrition education, stress management, and organizational support may promote healthier eating, improve mental wellbeing, and enhance self-perceived work performance in alignment with Saudi Vision 2030.

## Introduction

Nutrition is a central determinant of health and self-perceived work performance, and poor dietary practices are consistently associated with adverse outcomes and diminished occupational functioning (1). Among lifestyle-related risk factors, diet plays a particularly significant role in shaping preventable disease outcomes. The Global Burden of Disease Study reported that from 1990 to 2017, suboptimal diets contributed to nearly one in five deaths globally, more than any other behavioral factor. High sodium intake and insufficient consumption of fruits, vegetables, and whole grains were the leading contributors to this burden (2). A balanced diet ensures adequate macronutrients—carbohydrates, proteins, and fats—for energy, and micronutrients such as vitamins and minerals for immune defense, metabolic regulation, and cognitive function (3). Beyond nutrient quality, meal timing and eating regularity are also important, as irregular eating patterns are linked to obesity, weight gain, and metabolic disorders (4).

Workplace settings have become an important focus for nutrition promotion, as employee health directly influences organizational productivity. Wellness initiatives that offer nutrition education and access to healthier food options have shown positive effects on wellbeing and efficiency (5). Conversely, obesity among employees contributes to absenteeism, increased healthcare costs, and a greater risk of workplace injuries (6, 7). University employees, despite working in academic environments, are not exempt from these challenges. For some, stress and inactivity lead to overconsumption of calorie-dense foods. Such behaviors echo findings among university students, where stress and heavy workloads were linked to fast food reliance and sedentary lifestyles (8).

In Saudi Arabia, obesity and non-communicable diseases remain pressing public health concerns with major economic costs (9). This issue is highly relevant to the national Vision 2030 agenda, which prioritizes population health as a foundation for sustainable growth (10). Despite this priority, there is limited research examining the interplay between diet, nutrition knowledge, and stress in Saudi workplaces, particularly in higher education institutions. University employees represent a high-risk group due to irregular schedules, long working hours, and competing responsibilities. These factors foster behaviors such as meal skipping, late-night eating, and dependence on fast food, all of which increase risks of obesity, nutrient deficiencies, and gastrointestinal issues (11).

Nutrition knowledge influences eating behaviors by shaping food choices, portion control, and the ability to interpret nutrition labels. Higher nutrition literacy has been linked to healthier dietary practices and lower risks of non-communicable diseases (12). However, knowledge alone does not guarantee healthy behavior. Even individuals with higher education may struggle to distinguish between nutrient-rich and calorie-dense foods, particularly when workplace constraints and convenience override informed decision-making (13). In Saudi Arabia, myths and misconceptions about nutrition remain prevalent among adults, suggesting that access to information alone is insufficient (14). National surveys in Saudi Arabia confirm poor dietary trends among adults, including low fruit and vegetable intake and increased reliance on high-calorie foods (15). Moreover, irregular meal times have been associated with cognitive decline and reduced productivity (4). Dietary habits in this study were assessed using the validated Arabic Food Frequency Questionnaire, which provides reliable data on eating behaviors in working populations (16).

Stress is another major factor influencing nutrition and health outcomes among employees. Stressful experiences can disrupt normal eating patterns: some individuals cope by consuming calorie-dense, sugary, or fatty foods, while others under-eat or skip meals (17). For university employees, work-related stressors such as heavy teaching loads, administrative responsibilities, and difficulties balancing work and personal life exacerbate vulnerability to poor dietary habits (18). Chronic stress has broader implications, being associated with obesity, cardiovascular disease, and mental health issues such as depression (19). In Saudi Arabia, job stress has become a growing occupational health concern, closely linked to reduced productivity and absenteeism (20). Therefore, assessing stress alongside dietary habits and nutrition knowledge is essential for a holistic understanding of employee health. This study employed the Arabic version of the Stress Overload Scale, a validated tool for workplace contexts (21).

While global evidence highlights these associations, empirical research specific to Saudi university employees remains limited. Accordingly, two research questions guided the study, (1) What is the relationship between dietary habits, nutrition knowledge, and stress levels among employees? and (2) What perceptions, challenges, and barriers do employees face in relation to their nutrition and overall health? Targeted workplace interventions that build practical nutrition knowledge may help address this gap and promote sustainable behavior change. In this study, nutrition knowledge was measured using the General Nutrition Knowledge Questionnaire, a validated tool for assessing awareness of dietary guidelines, nutrient content, and food choice (22).

## Methods

This study employed a convergent parallel mixed-methods design, in which quantitative and qualitative data were collected concurrently during the same data collection period (September–October 2025), analyzed independently, and integrated at the interpretation stage. This design was selected because the study aimed simultaneously to quantify statistical associations among dietary habits, nutrition knowledge, and stress (quantitative strand), and to explore employees’ lived experiences, perceived barriers, and facilitators of healthy eating in the workplace (qualitative strand). A convergent design was preferred over explanatory sequential or exploratory sequential alternatives, as neither strand was intended to inform the development or explanation of the other; rather, both were designed to complement each other through simultaneous and independent inquiry, with integration occurring at the level of interpretation to provide a more complete and nuanced understanding of the phenomenon. Data were collected through an online survey and virtual in-depth semi-structured interviews, ensuring accessibility and broad reach.

### Sampling and data collection

Inclusion criteria included full- and part-time Saudi university employees, such as faculty, researchers, administrators, technicians, and assistants. Eligible participants were aged 18 or older and employed within Saudi universities. Exclusion criteria were employment outside Saudi universities or age below 18. For the qualitative component, purposive sampling was employed to ensure maximum variation across three theoretically meaningful dimensions known to shape workplace dietary experiences: (a) geographic region, (b) sex, and (c) occupational role (faculty member vs. administrator). This approach was selected not for statistical representativeness, but to achieve depth and diversity in exploring employees’ lived experiences of workplace nutrition. This is consistent with the aims of reflexive thematic analysis (Braun and Clarke, 2021) and the principle of information power, whereby sample adequacy is determined by the richness and relevance of data rather than numerical size. This strategy ensured diversity and allowed an in-depth exploration of workplace nutrition challenges and intervention feasibility. Data collection occurred between September and October 2025.

The study was conducted across universities in Saudi Arabia, spanning five geographic regions (Central, Northern, Southern, Eastern, and Western), covering both governmental and private institutions. The study did not seek to identify the number or names of participating universities for several reasons. First, withholding institutional identifiers reduces the risk of response bias inherent in self-administered surveys. Second, it eliminates social desirability bias among interview participants. Third, it protects participant confidentiality by preventing identification through institutional affiliation. The study focused on workplace environments as primary contexts shaping employees’ dietary behaviors, nutrition knowledge, and stress levels.

### Relationship between quantitative and qualitative samples

The qualitative participants were recruited as a nested sub-sample from within the quantitative survey respondents. At the end of the online survey, participants were invited to indicate their willingness to take part in a follow-up virtual interview. Nine individuals were then purposively selected from those who expressed interest, based on the variation criteria described above. This nested design ensures that both strands are grounded in the same target population, supporting meaningful integration of findings at the interpretation stage, consistent with a convergent parallel mixed-methods approach.

### Recruitment method

Participants were recruited via university emails, meetings, and social media. A survey link was provided, with an option to join interviews. Purposive sampling was select interviewees, who received study details and provided informed consent.

### Ethics approval and considerations

Ethical approval was obtained from the Local Committee of Bioethics (LCBE) at the Local Committee of Bioethics (HAP-09-A-043) at Northern Border University, Arar, Saudi Arabia, no. (77/25/H) during its 14th meeting for the academic year 2025, and all methods were performed in accordance with the relevant guidelines and regulations. Informed consent was secured from all survey participants, with additional electronic written consent collected from those who participated in virtual interviews. To maintain ethical rigor, each interview began with a confidentiality statement, and participants were reminded that discussions would be recorded. While no direct risks were anticipated, the research team remained attentive to workplace context and ensured respect for participants’ cultural, social, and personal beliefs. Confidentiality was preserved by storing all data securely on the Northern Border University OneDrive account, accessible only to the research team. Ethical considerations were embedded throughout data collection and analysis to ensure integrity and safeguard participant rights.

### Quantitative analysis approach

Data analysis was conducted using IBM SPSS v26. Descriptive statistics, including means, standard deviations, and frequency distributions, were calculated to provide an overview of the study variables. Associations between categorical variables were primarily examined using the chi-square test. Given that most variables were ordinal or could be treated as such, a linear-by-linear association approach was implemented to account for the ordered nature of the data, allowing detection of trends across increasing or decreasing levels. In cases where one of the variables was not ordinal, the standard Pearson chi-square test was applied. One-way ANOVA was used to compare group means. Polynomial contrasts were applied to assess linear and non-linear trends across ordinal categories. For group comparisons involving small cell sizes (*n* < 30), unweighted contrasts were prioritized to avoid undue influence of sample size on trend estimation, consistent with standard practice in ordinal trend analysis.

All statistical tests were conducted at a significance level of 0.05. Nutrition knowledge scores were calculated as the number of correct responses out of 85 questions (0–85), with a corresponding percentage scale for interpretability. Scores were categorized into four levels based on percentage values: low (0–24.99%), medium (25–49.99%), high (50–74.99%), and very high (75–100%). These cut-off points were established *a priori* to allow meaningful stratification of knowledge levels across participants and to enable comparison across demographic and behavioral subgroups in subsequent analyses. This categorization was applied consistently across all inferential analyses involving nutrition knowledge as a grouping variable. The sample size was determined using G*Power for two analyses. One-way ANOVA (stress/dietary behavior vs. knowledge) required 176 participants, while the chi-square test (stress vs. dietary behavior) required 174. To ensure sufficient power, the larger estimate of 176 was selected as the final sample size.

### Qualitative approach

#### Qualitative data collection

The study sought to recruit interview participants with varied job roles, genders, and work locations to enhance the depth and transferability of qualitative findings. A structured interview protocol was used to ensure consistency and reduce bias, giving all participants an equal opportunity to share their perspectives. Participants were informed they would receive a 50 SAR voucher in recognition of their time. Interviews were conducted virtually via Zoom (version 5.9.1), which reduced logistical challenges and distractions. Sessions were facilitated by a researcher with oversight from AMZ, an expert in qualitative interviewing, to ensure methodological rigor. Zoom is widely recognized as a reliable platform for qualitative data collection and an effective alternative to in-person interviews (23).

#### Interview analysis

Descriptive statistics were generated in Microsoft Excel (v16.98) to summarize demographic data. Interviews were analyzed using Braun and Clarke’s (2021) six-phase reflexive thematic analysis, focusing on both semantic and latent meanings to capture workplace experiences and challenges. Two researchers analyzed each transcript, incorporating peer debriefing and self-reflection to enhance rigor. Interviews were conducted virtually, audio-recorded, and transcribed verbatim with Transkriptor (v3.0.4). The adequacy of the sample size was supported by the information power model, ensuring data richness and alignment with qualitative research standards (Braun and Clarke, 2021). Sample adequacy was assessed using the information power model (32), which is more consistent with reflexive thematic analysis than traditional data saturation. Nine participants were judged sufficient based on: (1) a narrow, specific study aim; (2) a well-defined, homogeneous sample; (3) an established analytical framework (33); (4) rich 45–60 min interviews; and (5) a cross-case thematic analysis strategy. To protect participant confidentiality, all names presented in the qualitative results section are pseudonyms. Participants were informed of this procedure in the informed consent form, and this approach was approved by the institutional ethics committee.

Since the interviews were conducted in Arabic, participant quotes were translated into English and back-translated by two bilingual researchers to ensure accuracy. This methodological approach ensured credibility, cultural sensitivity, and depth in interpreting participant perspectives. To enhance trustworthiness, two researchers (FYA and AMA) independently conducted the first three analytical phases—familiarization, initial coding, and theme generation—before comparing their coding frames. Discrepancies in interpretation were resolved through structured discussion and consensus, with a senior researcher (AMZ) acting as arbiter where bilateral agreement could not be reached. Analytical memos documented all coding decisions and the resolution of interpretive disagreements, providing a transparent audit trail consistent with the principles of reflexive thematic analysis (Braun and Clarke, 2021). Researchers maintained analytical memos to document decisions during analysis. Initial codes included “peer effects,” “mealtime,” and “lack of access.” Using Braun and Clarke’s framework, FYA, or AMA with AMZ independently conducted the first three phases, while AMZ led reviewing, defining, and reporting themes, ensuring systematic, rigorous analysis.

## Results

### Quantitative approach

#### Sample characteristics and demographics

Overall, 232 participants fulfilled the inclusion criteria and completed the survey. Among the participants, 164 (70.7%) were females, 220 (94.8%) were Saudi nationals, and 157 (67.7%) were aged 25–44 years. Nearly half resided in the Northern region of Saudi Arabia (*n* = 111, 47.8%). Most participants were married (*n* = 163, 70.3%), while 52 (22.4%) were single; family size varied, with 20–30% distributed across categories of no children, 1–2, 3–4, or 5+. More than half (*n* = 135, 58.2%) were faculty members at Saudi universities, and work experience was evenly distributed across four categories (< 5, 5–10, 11–15, and 16 + years). Almost all participants (*n* = 224, 96.6%) worked mornings, with 164 (70.7%) working 6–8 h daily. Regarding health, 38 participants (16.4%) reported problems, the most common being diabetes mellitus (29.41%), thyroid disorders (26.47%), high blood pressure (26.47%), and asthma (14.71%). Additionally, 140 participants (60.3%) engaged in moderate physical activity, exercising 1–3 times per week (Table 1).

**Table 1:** Sample characteristics and demographics.

Variable	Categories	Frequency (*N* = 232)	Percentage%
Age group	Under 25	22	9.5
	25–34	70	30.2
	35–44	87	37.5
	45–54	44	19.0
	55 years and older	9	3.9
Gender	Male	68	29.3
	Female	164	70.7
Nationality	Saudi	220	94.8
	Non-Saudi	12	5.2
Regions of Saudi Arabia	Central	38	16.4
	Northern	111	47.8
	Southern	8	3.4
	Eastern	27	11.6
	Western	48	20.7
Marital status	Single	52	22.4
	Married	163	70.3
	Divorced	13	5.6
	Widowed	4	1.7
Number of children	0	68	29.3
	1–2	48	20.7
	3–4	67	28.9
	5+	49	21.1
Occupation	Faculty member	135	58.2
	Academic researcher	11	4.7
	Administrator	41	17.7
	Technician or administrative assistant	29	12.5
	Other	16	6.9
Experience (years)	<5	61	26.3
	5–10	57	24.6
	11–15	53	22.8
	>15	61	26.3
Daily work hours	<6	35	15.1
	6–8	164	70.7
	>8	33	14.2
Work shift	Morning	224	96.6
	Evening	8	3.4
Health problems	Yes	38	16.4
	No	194	83.6
Physical activity level	Low (I rarely exercise)	70	30.2
	Moderate (I exercise 1–3 times per week)	140	60.3
	High (I exercise 4 or more times per week)	22	9.5

#### Dietary habits

A substantial proportion of participants (*n* = 88, 37.9%) reported not eating breakfast or doing so only occasionally, consistent with findings that most consumed fewer than three meals per day (*n* = 142, 61.2%). Meal skipping was common, with 49.1% (*n* = 114) reporting that they always skipped meals and 30.6% (*n* = 71) doing so sometimes, yielding a combined total of 79.7% (*n* = 185) who skipped meals at least occasionally. Regarding eating during work hours, 22% (*n* = 51) reported doing so regularly, 42.2% (*n* = 98) sometimes, and 35.8% (*n* = 83) rarely or never. Although the majority preferred eating at home (*n* = 198, 85.3%), eating out was also common, with 47% (*n* = 109) dining out once weekly and 37.5% (*n* = 87) two to four times weekly. Most participants (*n* = 193, 83.2%) reported deliberation before food choices, and nearly 70% read nutritional labels at least sometimes. Dietary intake showed that most consumed 1–3 servings of fruits (70.7%), 2–3 servings of vegetables (72.4%), meat/legumes (79.3%), dairy (71.4%), and sweets (62.5%) daily, though 26.7% reported not consuming fruits at all (Table 2).

**Table 2:** Dietary habits of the participants.

Question	Answer	Frequency (*N* = 232)	%
Do you eat breakfast?	Yes	144	62.1
	No	35	15.1
	Sometimes	53	22.8
How many meals do you eat per day?	<3	142	61.2
	3	80	34.5
	>3	10	4.3
Do you skip any meals per day?	Yes	114	49.1
	No	47	20.3
	Sometimes	71	30.6
Do you eat your main meals during work hours?	Yes, regularly	51	22.0
	Sometimes	98	42.2
	Rarely	45	19.4
	No	38	16.4
How often do you eat out?	I do not eat out	27	11.6
	Once a week	109	47.0
	2–4 times a wee	87	37.5
	Daily	9	3.9
Do you spend a lot of time thinking before choosing a food when you are hungry?	Yes	103	44.4
	No	39	16.8
	Sometimes	90	38.8
Which one do you prefer?	Eating at home	198	85.3
	Eating from outside	34	14.7
Do you read the nutritional labels on the back of products?	Yes	67	28.9
	No	72	31.0
	Sometimes	93	40.1
How many servings of fruit do you consume per day?	No, I do not eat fruits	62	26.7
	1–3	164	70.7
	4–5	6	2.6
How many servings of vegetables do you consume per day?	Never	26	11.2
	1	7	3.0
	2–3	168	72.4
	4–5	29	12.5
	> 5	2	0.9
How many servings of meat and legumes do you consume per day?	Never	21	9.1
	1–3	184	79.3
	4–5	20	8.6
	> 5	7	3.0
How many servings of dairy do you consume per day?	Never	43	18.5
	2–3	145	62.5
	4–5	40	17.2
	> 5	4	1.7
How many servings of sugars and sweets do you consume per day?	Never	36	15.5
	2–3	138	59.5
	4–5	48	20.7
	> 5	10	4.3
How many servings of starches do you consume per day?	Never	20	8.6
	2–3	136	58.6
	4–5	57	24.6
	> 5	19	8.2
How many glasses of water do you consume per day?	2–3	83	35.8
	4–6	109	47.0
	> 6	40	17.2

#### Opinion regarding the role of nutrition and its impact on health and job performance

**Figure 1:**
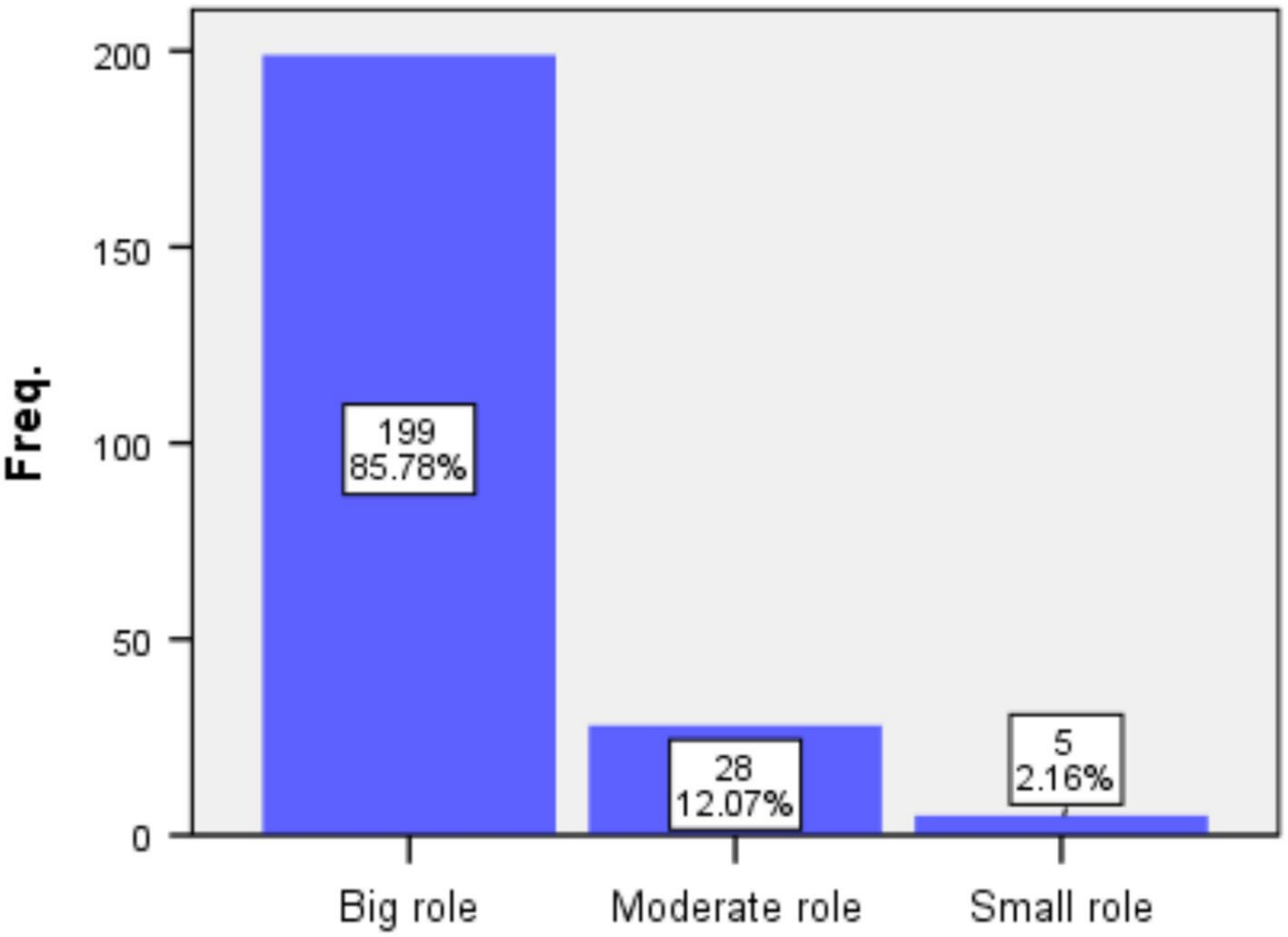
Participants’ opinion regarding the role of nutrition and its impact on health and job performance.

The majority of the participants (*n* = 199, 85.78%) perceived nutrition as having an important role in their health and job performance, reflecting a subjective belief rather than an objectively measured outcome (Figure 1).

#### Response to stress overload scale

**Figure 2:**
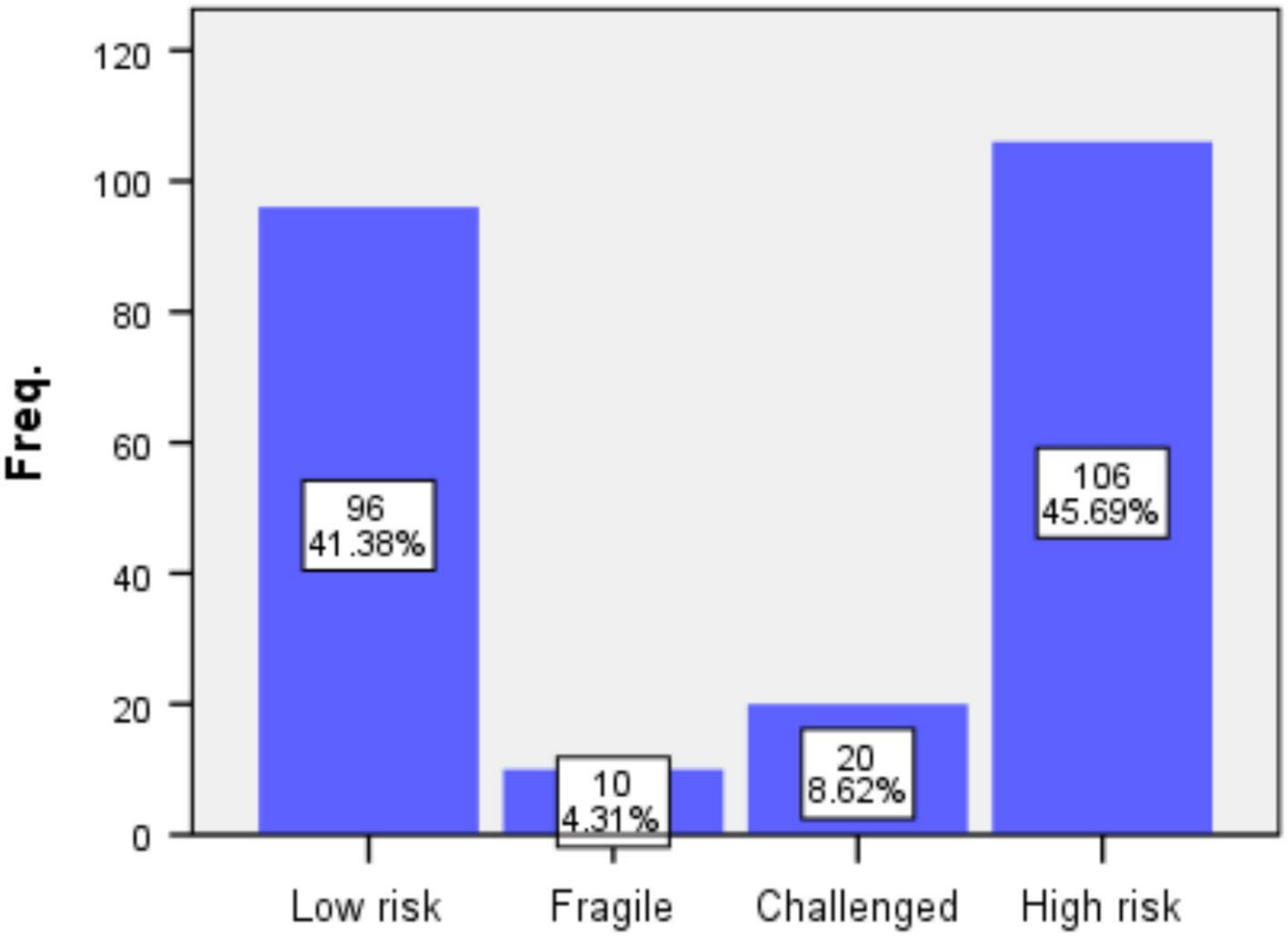
Categorizing participants according to their Stress Overload Scale (SOS)-scores.

The overall Event Load (EL) mean was 3.57, indicating a tendency toward high perceived load. Seven items scored between 3 and 3.5, reflecting frequent agreement with feelings of being rushed or burdened, whereas five items approached 4, showing strong agreement with being overcommitted, strained, or having too much to do. Personal vulnerability (PV) had an overall mean of 3.19, suggesting moderate levels (Supplementary Table 1). Most items averaged 3–3.5; however, “unconfident” was lower (2.21), while “like you couldn’t cope” was higher (3.84). Items outside EL/PV varied: “interested” and “carefree” averaged 2.5–3 (more disagreement), “focus on important things” and “at peace” scored 3–3.5 (moderate agreement), and “calm” and “generous” approached 4, reflecting stronger agreement. Figure 2 shows that the distribution of participants according to their level of stress is kind of bi-modal, where participants are roughly either at high-risk of stress (*n* = 96, 41.38%) or at low-risk of stress (*n* = 106, 45.69%), with few of them are in-between (i.e., in the “fragile” or “challenged” levels of stress).

#### Response to knowledge questions

The study revealed considerable variation in participants’ nutrition knowledge. Most participants (≥60%) demonstrated correct knowledge regarding the need to consume more vegetables (63.4%) and water (75.9%), and to limit foods and drinks with added sugars (67.2%). Similarly, 62.1% recognized the need to reduce trans fats, while 64.7% correctly rejected the misconception that all fats should be avoided for weight control. Knowledge was also strong regarding food composition: most participants knew that yogurt is low in added sugar (78%), ice cream is high in sugar (74.6%), canned soup is high in salt (65.1%), oatmeal is high in fiber (73.7%), and poultry is a good protein source (84.1%). Participants also distinguished well between starchy and non-starchy foods (≥70%). Furthermore, the majority recognized healthy eating behaviors such as not eating while watching TV (75%), reading food labels (78%), monitoring food intake (79.3%), and weight control (75%) (Supplementary Tables 2.1.1–2.1.9).

**Figure 3:**
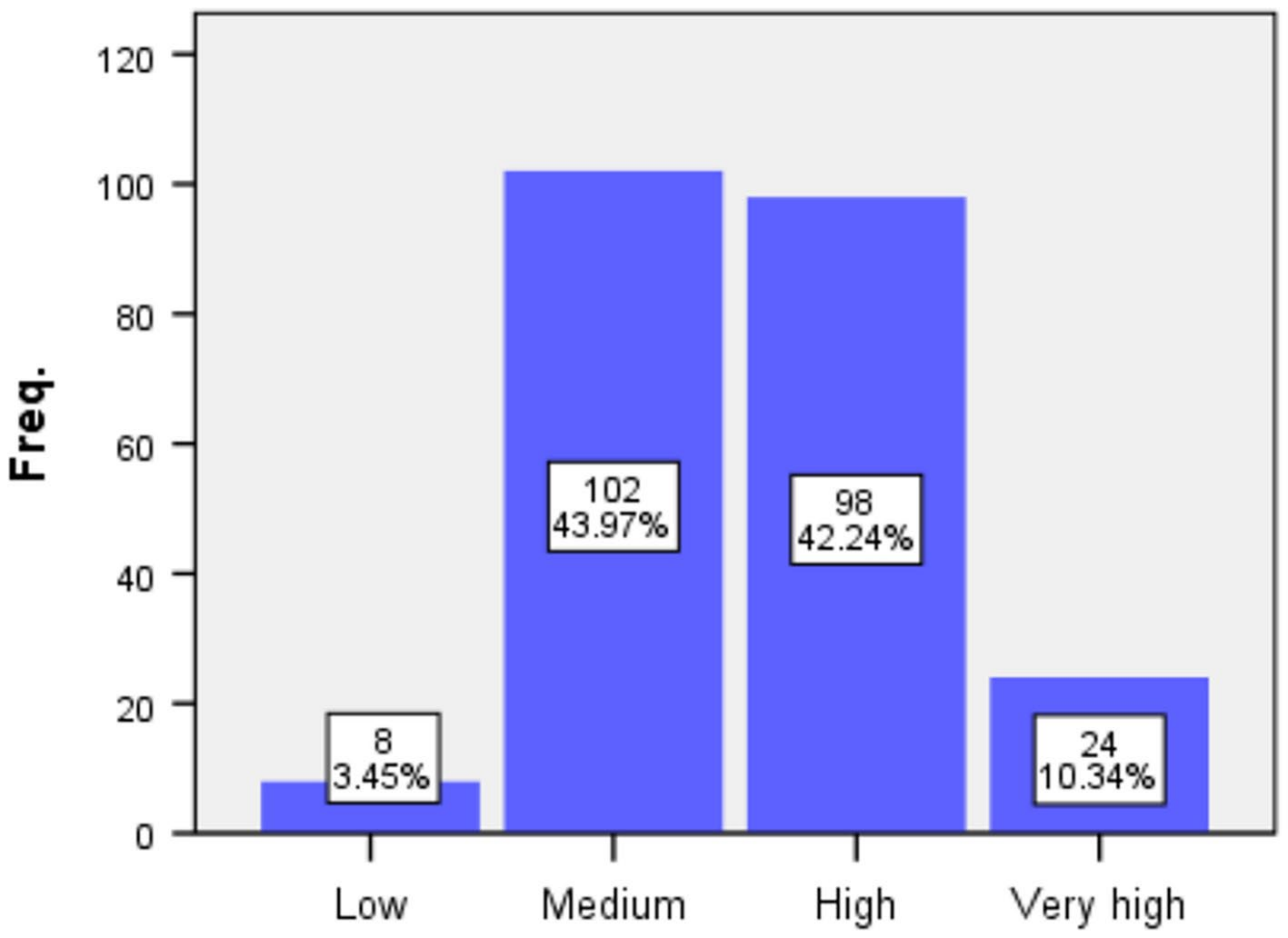
Classification of participants according to their knowledge scores.

Moderate knowledge (40–59%) was observed for identifying healthier food choices at restaurants, calcium equivalence in milk types, recommended dairy products, and links between diet and heart disease. Less than 40% correctly identified main fat types (e.g., butter as saturated fat, olive oil as monounsaturated), foods high in trans fats, cooking methods requiring added fat, or the recommended proportion of starchy foods. Alarmingly, very few participants recognized expert guidance on daily fruit and vegetable servings (13.4%) or the healthiest dessert choice (12.9%). Overall, participants showed relatively strong knowledge of basic nutrition concepts and food classification but had gaps in applied dietary recommendations, nutrient-specific details, and disease-related nutrition links. These findings highlight the need for targeted nutrition education programs focusing on practical dietary recommendations (e.g., fruit/vegetable servings, fat types, and disease prevention strategies) to improve translation of nutrition knowledge into healthier behaviors (Supplementary Table 2.2). The mean nutrition knowledge score was 44.7 out of 85 (SD = 14.1), equivalent to 52.6% (SD = 16.6) on a percentage scale. The majority of participants (*n* = 200, 86.2%) fell within the medium or high knowledge categories (Figure 3).

#### The association between dietary habits and stress

Several dietary behaviors showed significant associations with stress (*p* < 0.05). Breakfast consumption was strongly linked, with high stress risk increasing as frequency decreased: 37.5% among those who always/usually ate breakfast, 56.6% among those who sometimes did, and 62.9% among those who never did. Similarly, meal frequency mattered, as participants eating fewer than three meals daily were more likely to be at high risk of stress (51.4%) compared to those consuming three meals (33.8%). Skipping meals was also associated with greater stress risk (50% vs. ~40% among non-skippers). Fruit and vegetable intake demonstrated protective effects: non-consumers were more likely to report high stress (59.7 and 69.2%, respectively) compared to those consuming daily servings (40.2% for fruits; 27–47% for vegetables). Finally, water consumption showed an inverse association, with stress risk decreasing from 55.4% among those drinking 2–3 cups to 37.5% among those drinking more than six cups daily (Supplementary Table 3).

#### The association between dietary habits and nutrition knowledge

Nutrition knowledge scores were significantly associated with several dietary behaviors (*p* < 0.05). Participants who ate main meals during work had lower nutrition knowledge scores (~50%) compared to those who never did (>55%). Reading nutrition labels was positively linked to knowledge, with nutrition knowledge scores decreasing from 54.91% (usually) to 49.1% (never). Daily fruit and vegetable consumption was associated with higher nutrition knowledge scores (>50%), while non-consumers scored lower (48.62% for fruits; <50% for vegetables). Starch intake also mattered, with non-consumers scoring lowest (38.35%) versus 51–60 among consumers. Water intake showed a positive gradient, increasing from 49.11% (2–3 cups/day) to 56.24% (>6 cups/day) (Supplementary Table 4).

#### The association between nutrition knowledge and stress

Stress levels were significantly associated with knowledge (*p* < 0.05). Participants at high risk of stress had lower average knowledge scores (49.42%), compared to those at lower risk, whose scores generally exceeded 55%. This indicates an inverse relationship between stress and nutritional knowledge (Table 3).

**Table 3:** The association between nutrition knowledge and stress.

Stress level	*N*	Nutrition knowledge score		*p*-value
		Mean	SD	
Low risk	96	56.19	16.83	0.009
Fragile	10	41.18	8.80	
Challenged	20	58.12	19.55	
High risk	106	49.42	15.28	

#### The association between demographic variables and stress

Work experience was significantly associated with stress (*p* < 0.05). Participants with less than 5 years of experience had a higher likelihood of high stress (62.3%) compared to those with greater experience, whose stress risk ranged between 34 and 43%, indicating reduced stress with increased work experience (Supplementary Table 5).

#### The association between other variables and nutrition knowledge

Regions of Saudi Arabia and opinions on nutrition’s role in job performance were significantly associated with knowledge (*p* < 0.05). Participants from the Western region had higher average nutrition knowledge scores (64.71%) than other areas (44–54%). Likewise, stronger belief in nutrition’s impact corresponded to higher nutrition knowledge scores (53.63% vs. 47.94 and 38.35%) (Supplementary Table 6).

#### Other associations

Regions of Saudi Arabia were not significantly associated with physical activity levels (*p* > 0.05). Daily work hours were also not significantly linked to the frequency of eating main meals at work (*p* = 0.074), though those working <6 h/day were somewhat more likely to eat at work (34.3% vs. ~20%). In contrast, the number of children was significantly associated with eating out (*p* < 0.05); participants with no or few children rarely ate out (<6%), whereas those with 3–4 or more children had higher likelihoods (16.4 and 24.5%, respectively) (Supplementary Tables 7.1, 7.2).

### Qualitative approach

#### Participant characteristics

Nine participants who completed the survey took part in 45–60 min in-depth, semi-structured virtual interviews. Among them, seven (77.8%) were faculty members and two (22.2%) were administrators. Participants were primarily from the Northern region of Saudi Arabia (*n* = 6, 66.7%), with two (22.2%) from the Eastern region and one (11.1%) from the Western region. The majority were female (*n* = 8, 88.9%), and one (11.1%) was male. Regarding educational attainment, seven participants (77.8%) held doctoral degrees, while two (22.2%) held bachelor’s degrees as their highest qualification.

#### Qualitative analysis themes

The themes that emerged from qualitative analysis were workplace demands and environmental constraints shape dietary behaviors, interconnection of nutrition, energy, and mental Health in the workplace, sociocultural, economic, and workplace factors shaping dietary choices, and knowledge, awareness, and access as determinants of healthy eating.

### Theme 1. Workplace demands and environmental constraints shape dietary behaviors

Work demands and the work environment strongly influence employees’ dietary habits as reported by study participants. Participants emphasized that knowledge of healthy nutrition does not always translate into practice. Nawal (administrator) explained, “Even when we know better, hunger during breaks pushes us toward quick fixes—often fast food.” Sarah (faculty member) further noted that workload and limited workplace options shape dietary behavior. Tala (faculty member) noted that being busy greatly impacts eating behaviors:

Honestly, being busy greatly impacts your eating habits. When work consumes you, making a salad or buying something healthy feels impossible. You end up grabbing whatever is near, and the busier you are, the harder mindful food choices become.

Similarly, Nawal (administrator) observed the unstructured nature of work hours, stating, “I may start with breakfast at home, but at work, time feels unstructured—between 10 a.m. and 1 p.m., it’s just coffee and sweets. Habits like these reveal how damaging unhealthy work routines can be.” Moreover, participants described how dense schedules and demanding shifts disrupt eating patterns by increasing stress and reducing appetite. For example, Rana (faculty member) described “when I’m in a hurry, I eat little—even if my body’s needs aren’t met.” Participants also shared a list of challenges that they face in maintaining healthy eating habits (Table 4).

**Table 4:** Challenges employees face in maintaining healthy eating habits.

Challenge and Description
Limited healthy food access—Few nutritious options on campus, with cafeterias often dominated by snacks like chips and chocolate, forcing reliance on fast food or deliveries
Inadequate facilities—Lack of cafeterias, kitchen appliances, or designated areas restricts employees from preparing or storing balanced meals.
Time constraints—Back-to-back lectures, meetings, and long working hours leave little opportunity for regular meals or hydration
Workload and fatigue—Heavy responsibilities drain energy and reduce the motivation to prioritize proper eating.
Stress-related disruptions—Work-related stress contributes to irregular eating, skipping meals, or choosing high-calorie comfort foods.
Social pressures—Workplace events and group eating norms often push individuals to consume unhealthy foods out of courtesy or to avoid embarrassment.
Repetitive, calorie-dense options—Limited variety in available foods leads to dependence on high-calorie meals, reducing control over diet quality.
Forgetting meals—Busy schedules frequently cause employees to miss meals, sometimes relying on device reminders to eat or drink.

### Theme 2: interconnection of nutrition, energy, and mental health in the workplace

Participants were asked to share their thoughts on how nutrition, energy, and mental health intersect. Tala (faculty member) explained that good nutrition provides the energy a person needs while supplying essential vitamins and nutrients that support overall vitality. Nawal (administrator) described the immediate impact of skipping breakfast, stating, “Missing breakfast drains my energy and makes it hard to function all day.” Participants also discussed the importance of meal content, emphasizing that both quality and quantity matter. Noor (faculty member) shared.

When I eat fast food or meals high in fat, I feel drained and lack energy to socialize. Consuming too much oil, meat, or sugar affects my mood and appearance, sometimes causing pimples that make me self-conscious and hesitant to interact with others.

Nutrition was also linked to mental health. Nawal reported experiencing severe psychological exhaustion, including social withdrawal and heightened sensitivity to others, and expressed surprise that a lack of vitamins could contribute to such emotional challenges. Other participants perceived improvements in mental wellbeing with healthier habits or weight loss. Tala explained.

Being busy at work has a major impact on my eating habits. I rarely think about preparing a salad or buying something healthy. Instead, I grab whatever is quick and convenient. The busier I get, the harder it becomes to make mindful food choices.

Overall, participants highlighted that nutrition affects not only physical energy but also mental health and social functioning, and that workplace demands significantly shape food choices and overall wellbeing.

### Theme 3: sociocultural, economic, and workplace factors shaping dietary choices

Participants discussed various factors influencing their food choices, with gender emerging as a notable factor. Sanad, Nawal, and Rania shared the view that women typically have higher nutritional knowledge than men in Saudi universities. Sanad, a faculty member, explained, “Men often do not pay attention to their nutrition until after developing an illness.” Similarly, Sarah, a faculty member, observed that women are more likely to prepare their meals at home, whereas men usually do not. Economic status was also highlighted as an influencing factor. Rana, a faculty member, explained that she can afford regular vitamin checks and takes supplements to correct deficiencies.

Cultural factors were noted by Amal and Nawal, administrators at different workplaces. They reported that employees often feel obliged to partake in food shared during workplace occasions, even when it is unhealthy, to avoid embarrassment. Other common barriers included limited time, high workload, lack of nutritional resources, absence of standardized break times, and insufficient facilities to store or heat meals. These constraints contributed to unhealthier food choices. Rana illustrated these challenges, stating.

At my university, back-to-back lectures make it hard to eat properly. With limited breaks and no food options on campus, it’s easier to rely on chocolate or quick snacks for energy. The lack of time and available healthy food is a major challenge.

Participants emphasized the need for workplace support in Saudi universities to facilitate healthier eating habits, including better access to nutritious food, flexible break times, and resources for meal preparation (Table 5).

**Table 5:** Support needed by employees to improve healthy eating at Saudi universities.

Support and Description
Accessible healthy food—Affordable, nutritious options available in cafeterias, vending machines, or food outlets.
Quality and variety—Wider selection of balanced meals and snacks that are quick and convenient.
Designated eating spaces—Areas within the workplace for comfortable and proper mealtimes.
Institutional support—Resources, programs, and policies that promote healthy eating, including nutrition education.
Financial support—Food allowances or subsidies to make healthier choices more affordable.
Social encouragement—A supportive culture where colleagues respect and encourage healthy food choices without judgment.
Practical guidance—Simple, home-based diet plans and easy-to-understand information about healthy meals suited to work settings.
Integrated health programs—Initiatives that combine nutrition awareness with psychological support, physical activity (e.g., walking campaigns), and cafeteria improvements.
Community-wide education—Awareness and training for students, faculty, and food providers to ensure healthier options are consistently available.

### Theme 4: knowledge, awareness, and access as determinants of healthy eating

Participants were asked to define good nutrition and its relationship to health. Many, including Sanad, a faculty member, summarized it in a single word: “balance.” Several emphasized the need to raise awareness about healthy eating and dispel misconceptions that nutritious food is bland or restrictive. As Amal (an administrator) explained:

The most important goal is to help people understand that healthy nutrition is not about deprivation or bland food. By controlling portions and including fruits, vegetables, and healthy snacks, people can enjoy nutritious meals that support overall health, not only weight loss.

Participants noted that nutrition knowledge is limited among university employees, and administrators should promote awareness while facilitating access to healthier food options on campus. Awareness of health risks from excessive sugar and unhealthy fats also unconsciously reduces consumption, as Rania, a faculty member, shared. Sanad observed that employees specialized in nutrition tend to have deeper knowledge and are more likely to follow healthy dietary habits. However, knowledge alone does not always prevent unhealthy choices, as Rania also noted, and the desire to eat healthily does not always translate into practice, as reported by Fooz, a faculty member. Participants were further asked to provide dietary advice for new university employees, summarized in Table 6.

**Table 6:** Nutrition advice shared by participants for new employee.

Advice and Description
Bring meals from home or order from healthy restaurants to avoid relying on unhealthy workplace options.
Avoid peer pressure by limiting eating with colleagues who have unhealthy habits.
Start the day right with a balanced breakfast and avoid beginning the day with sweets.
Adopt a healthy lifestyle that balances nutrition, adequate sleep, and regular physical activity.
Maintain a consistent eating routine that supports energy, focus, and productivity.
Prioritize long-term health, recognizing that good nutrition enhances performance and prevents burnout.
Stay mindful of workplace eating culture and avoid indulging in unhealthy foods out of courtesy or social pressure.
Make gradual changes to eating habits rather than abrupt shifts for sustainability.

#### Integrated findings: joint display of quantitative and qualitative results

Table 7 presents an integration of the key quantitative findings with corresponding qualitative themes and participant quotes, yielding meta-inferences that neither strand could produce independently.

**Table 7:** Joint display: integration of quantitative and qualitative findings.

Quantitative finding	Statistical result	Qualitative theme	Supporting participant quote	Meta-inference
Irregular breakfast associated with higher stress	High stress: 62.9% (never ate breakfast) vs. 37.5% (always/usually ate breakfast); *p* < 0.05	Theme 1: Workplace Demands and Environmental Constraints Shape Dietary Behaviors	*“Missing breakfast drains my energy and makes it hard to function all day”*—Nawal, administrator	Workplace time pressures disrupt breakfast habits and elevate stress; structural interventions such as protected mealtimes are needed alongside individual behavior change
Meal skipping associated with higher stress	High stress: 50% (skippers) vs. ~40% (non-skippers); *p* < 0.05	Theme 1: Workplace Demands and Environmental Constraints Shape Dietary Behaviors	Workplace Demands and Environmental Constraints Shape Dietary Behaviors*”When I’m in a hurry, I eat little—even if my body’s needs aren’t met”*—Rana, faculty member	Occupational demands structurally impose meal skipping; this cannot be resolved through nutrition education alone without organizational-level change
Low fruit intake associated with Higher stress	High stress: 59.7% (non-consumers) vs. 40.2% (daily consumers); *p* < 0.05	Theme 3: Sociocultural, Economic, and Workplace Factors Shaping Dietary Choices	*“With limited breaks and no food options on campus, it is easier to rely on chocolate or quick snacks for energy”*—Rana, faculty member	Limited campus food access structurally constrains fruit consumption independent of knowledge or intention
Low vegetable intake associated with higher Stress	High stress: 69.2% (non-consumers) vs. 27–47% (daily consumers); *p* < 0.05	Theme 4: Knowledge, Awareness, and Access as Determinants of Healthy Eating	*“The desire to eat healthily does not always translate into practice”*—Fooz, faculty member	The intention–action gap is compounded by environmental barriers; knowledge-based interventions alone are insufficient
Higher nutrition knowledge associated with lower stress	Mean knowledge: 56.19% (low-risk stress, *n* = 96) vs. 49.42% (high-risk stress, *n* = 106); *p* = 0.009	Theme 4: Knowledge, Awareness, and Access as Determinants of Healthy Eating	*“Awareness of health risks from excessive sugar and unhealthy fats also unconsciously reduces consumption”* — Rania, faculty member	Nutrition knowledge may act as a protective factor against stress through dietary self-regulation; knowledge-building should be a component of workplace wellness programs

## Discussion

This study examined the relationship between nutrition, stress, and job performance among university employees. Healthier dietary habits and higher nutrition knowledge were associated with lower stress and better self-perceived work performance, while irregular eating and meal skipping were associated with higher stress levels. Notably, 60.3% of participants reported engaging in moderate physical activity at least three times per week, contrasting with a study reported that 52.1% of Saudi women in office environments were insufficiently active. Factors such as longer working hours, older age, and private-sector employment were associated with lower activity levels (24). This discrepancy may reflect differences in workplace structure, institutional health promotion efforts, or access to fitness facilities, underscoring the role of organizational context in shaping employee health behaviors.

Despite participants’ moderate-to-high nutrition knowledge, substantial gaps were evident. Most participants correctly identified the importance of limiting added sugars (67.2%), increasing water intake (74.9%), and consuming more vegetables (63.4%), with 62.1% recognizing the need to reduce trans fats. These findings differ from a study reported inadequate nutrition knowledge among adults across four Arab countries despite basic conceptual understanding. Variations may be due to differences in educational attainment, exposure to nutrition-related media, or the measurement instruments employed (25). Bany-Yasin et al. emphasized that awareness alone does not equate to comprehensive understanding, highlighting the need for targeted educational initiatives. Similarly, a study found that higher nutrition literacy among white-collar employees was positively associated with healthier dietary behaviors and a greater tendency to seek nutrition information (26). Together, these studies suggest that enhancing both individual knowledge and supportive environments can foster healthier dietary practices among employees.

Meal frequency emerged as a critical determinant of dietary quality and workplace functioning. Most participants reported consuming fewer than three meals per day and frequently skipping breakfast. This aligns with a cross-sectional study found that habitual breakfast skipping is associated with lower diet quality, increased consumption of energy-dense, nutrient-poor foods, and overall dietary imbalance (27). The present study reinforces the importance of regular, balanced meals, particularly breakfast, for sustaining energy, cognitive focus, and nutritional adequacy in occupational settings.

Participants’ perceptions emphasized the role of nutrition in overall health and work performance, with 85.78% acknowledging its significance. This is consistent with a study found that workplace interventions targeting nutrition and physical activity improved employee health, reduced absenteeism, and enhanced productivity (28). As noted by Grimani et al., the effectiveness of such programs depends on factors including intensity, adherence, and organizational culture (28). Additionally, work experience was significantly associated with stress levels, as participants with fewer than 5 years of tenure reported higher stress than those with longer experience. This observation aligns with another study reported that less experienced lecturers face greater occupational stress, likely due to fewer coping mechanisms, lower professional confidence, and limited familiarity with institutional processes (29).

Nutrition knowledge was also positively correlated with specific dietary behaviors, including reading food labels. However, this contrasts a study observed limited engagement with labeling despite positive attitudes, reflecting a common gap between knowledge and practice (30). These findings emphasize that awareness alone may be insufficient to drive behavioral change. Importantly, the implications align with national health priorities outlined in Saudi Vision 2030, particularly preventive healthcare and promotion of healthy lifestyles. Recent evidence highlights psychological benefits of nutrition education; for instance, a study found that improved nutrition knowledge among young Saudi adults significantly reduced anxiety, demonstrating mental health advantages (31). Similarly, the present study indicates that higher nutrition knowledge is associated with lower stress and improved self-perceived occupational performance, consistent with existing evidence linking nutrition awareness and mental wellbeing.

Integrating findings from both strands yields three overarching meta-inferences (Table 7). First, workplace structural factors—not individual knowledge deficits—are the primary drivers of poor dietary behaviors and elevated stress. Meal skipping, irregular breakfast, and low fruit and vegetable intake were each significantly associated with higher stress risk (*p* < 0.05), while Theme 1 reveals these behaviors are structurally imposed through demanding schedules, absent eating spaces, and limited healthy food access. Dietary interventions must therefore target organizational and environmental conditions alongside individual behavior. Second, nutrition knowledge is necessary but insufficient as a standalone protective factor. Although higher knowledge was significantly associated with lower stress (*p* = 0.009) and healthier behaviors, Theme 4 highlighted a consistent intention–action gap, suggesting future interventions must combine nutrition education with structural enablers including affordable healthy food and flexible break schedules. Third, the relationship between diet and stress is bidirectional. Poor dietary habits were associated with higher stress quantitatively, while Theme 2 reveals that stress itself disrupts eating patterns, creating a self-reinforcing cycle. Workplace wellness programs should therefore address nutrition and stress management simultaneously.

### Strengths, limitations, and future directions

This study demonstrates several methodological strengths, including the use of validated Arabic instruments to ensure cultural relevance and psychometric reliability, and the application of Braun and Clarke’s six-phase framework to enhance qualitative rigor. The mixed-methods design enabled a comprehensive examination of relationships among nutrition knowledge, dietary behaviors, and stress, while the inclusion of participants from diverse demographic and occupational backgrounds increased contextual applicability. However, limitations should be considered. The quantitative sample was recruited through voluntary convenience sampling via university email lists, staff meetings, and social media, without random selection, which may limit the generalizability of findings to other university employee populations in Saudi Arabia and beyond. The cross-sectional design prevents causal inference, and reliance on self-reported data may introduce recall and social desirability biases. Additionally, excluding individuals with limited internet access or Arabic literacy may have affected representativeness. Participating institutions were not named or enumerated to reduce response bias in the self-administered survey, minimize social desirability among interview participants, and protect participant confidentiality through de-identification of institutional affiliation. Future studies may consider multi-site designs with formal institutional consent and site-level reporting where ethical and logistical conditions permit.

The qualitative sample mirrors the quantitative sample demographically, with both skewing toward female participants, faculty members, and Northern region employees. Consequently, perspectives of male employees, administrators, and other regions are less represented. Future studies should ensure greater demographic balance to strengthen transferability. Moreover, transferability is bounded to the Saudi university workplace context. Applicability to other settings may be assessed based on the study’s thick description of context, participants, and procedures. Future research across wider institutions, regions, and demographic groups would strengthen transferability. The quantitative sample (*n* = 232) is relatively small and self-selected, which may limit the generalizability of findings to other university employee populations in Saudi Arabia and beyond. Despite these limitations, the study offers valuable preliminary insights and underscores the need for longitudinal and intervention-based research. Future research should use longitudinal designs to assess the long-term impact of workplace nutrition initiatives on employee health, productivity, and job satisfaction. Studies should examine organizational influences, such as leadership and culture, and evaluate targeted, culturally appropriate strategies to inform sustainable, evidence-based workplace wellness interventions.

## Conclusion

This study highlights a potential association between dietary behaviors, stress, and work performance among university employees. Healthier eating patterns and higher nutrition knowledge scores were associated with lower stress levels and better self-perceived work performance, while irregular meal patterns were associated with poorer outcomes. Despite general nutrition awareness, knowledge gaps underscore the need for workplace nutrition education. Further large-scale and intervention-based studies are needed to confirm these findings and inform evidence-based initiatives aligned with Saudi Vision 2030.

## Data Availability

The original contributions presented in the study are included in the article/Supplementary material, further inquiries can be directed to the corresponding author/s.
