# Supplementary material for: Associations of nutrition knowledge and dietary habits with stress among university employees in Saudi Arabia: mixed-methods evidence
Source: Front Nutr. 2026 May 26;13:1834114. doi: 10.3389/fnut.2026.1834114 (PMC13246399; doi:10.3389/fnut.2026.1834114)
Supplement: Supplementary file 1 [file Table_1.DOCX]

Supplementary Materials

Results

**Supplementary Table 1. Response to Stress Overload Scale (SOS)**

| **Part** | **Item** | **Mean** | **SD** |
| --- | --- | --- | --- |
| EL | Strained | 3.78 | 0.97 |
|  | Overextended | 3.38 | 1.11 |
|  | Swamped by your responsibilities | 3.66 | 1.17 |
|  | That there wasn't enough time to get to everything | 3.45 | 1.21 |
|  | Like you were rushed | 3.47 | 1.17 |
|  | Like you had a lot on your mind | 3.99 | 0.99 |
|  | Overcommitted | 3.84 | 1.05 |
|  | Like things kept piling up | 3.42 | 1.23 |
|  | Like you had to make quick decisions | 3.43 | 1.13 |
|  | Like you didn't have time to breathe | 3.12 | 1.36 |
|  | Like you were carrying a heavy load | 3.42 | 1.19 |
|  | Like there was 'too much to do, too little time' | 3.59 | 1.15 |
|  | **Overall** | **3.55** | **0.89** |
| PV | Inadequate | 2.97 | 1.18 |
|  | Unconfident | 2.21 | 0.89 |
|  | No sense of getting ahead | 3.19 | 1.21 |
|  | That the odds were against you | 3.15 | 1.28 |
|  | Like you couldn't cope | 3.84 | 1.03 |
|  | Like nothing was going right | 3.43 | 1.23 |
|  | Powerless | 3.11 | 1.21 |
|  | Like your life was 'out of control' | 3.19 | 1.27 |
|  | Like asking 'what else can go wrong?' | 3.17 | 1.20 |
|  | Like things couldn't get worse | 3.16 | 1.14 |
|  | Like there was no escape | 3.38 | 1.09 |
|  | Like just giving up | 2.93 | 1.28 |
|  | **Overall** | **3.14** | **0.86** |
| Other (not related to  EL/ PV) | Calm | 3.59 | 1.03 |
|  | Interested | 2.59 | 1.13 |
|  | Generous | 3.99 | 0.79 |
|  | Carefree | 2.78 | 1.15 |
|  | Like you were able to focus on the important things | 3.48 | 1.12 |
|  | At peace | 3.50 | 1.05 |
|  | **Overall** | **3.32** | **0.61** |

EL: Event Load; PV: Personal Vulnerability

Main Findings:

Event Load (EL)

- The average response for 7 items (out of 12) is roughly in the range 3-3.5. In such items, usually the agreement (agree + strongly agree together) is more present than the disagreement, even if it does not constitute a majority. These items are:
- Like you didn't have time to breathe
- Overextended
- Like you were carrying a heavy load
- Like you had to make quick decisions
- Like things kept piling up
- Like you were rushed
- That there wasn't enough time to get to everything
- On the other hand, in 5 items (out of 12), the average response is approaching 4, which indicates that most of the participants expressed agreement. These items are:
- Like there was 'too much to do, too little time'
- Swamped by your responsibilities
- Strained
- Overcommitted
- Like you had a lot on your mind
- The overall EL-score has an average of 3.57, which shows some general tendency towards high EL (since the average approaches 4).

Personal Vulnerability (PV)

Most of the items under PV have an average response in the range 3-3.5 (which usually indicates that agreement is more than disagreement, even if it does not make a majority). However, some of the items showed different level of agreement. The item "unconfident" had an average response of 2.21 (which is approximately 2, i.e., indicating dominance of disagreement), and the items "like just giving up" and "inadequate" have an average response that's almost 3 (which indicates moderateness; i.e., balanced agreement-disagreement and/or a considerable presence of neutralism). On the other hand, the item "like you couldn't cope" has an average response of 3.84 (which is approximately 4, i.e., indicating dominance of agreement). Finally, because of the dominance of items with average response in the range 3-3.5, the overall PV-score has also an average which is in this range (specifically: 3.19), which indicates a general tendency towards moderateness in PV.

**Other (not Related to EL/ PV):**

Items under this category are varying in terms of level of participant agreement. Two items (namely: interested and carefree) have average response in the range 2.5-3 (which generally shows that disagreement is more than agreement, even if it's not a majority). On the other hand, two items (namely "like you were able to focus on the important things" and "at peace") have average response in the range 3-3.5 (which generally indicates that agreement is more than disagreement, even if it's not a majority), while two items ("calm" and "generous") have average response that's approaching / approximately 4 (which indicates the dominance of agreement).

**Supplementary Table 2.1.1. Response to Nutrition Knowledge Questions for Food Type and Recommendation**

| **Food Type** | **Recommendation N (%)** | | | |
| --- | --- | --- | --- | --- |
|  | **More** | **Balanced (equal)** | **Less** | **Not sure** |
| Fruit | **106 (45.7%)** | 109 (47%) | 12 (5.2%) | 5 (2.2%) |
| Foods and drinks with added sugar | 23 (9.9%) | 47 (20.3%) | **156 (67.2%)** | 6 (2.6%) |
| Vegetables | **147 (63.4%)** | 75 (32.3%) | 9 (3.9%) | 1 (0.4%) |
| Fatty foods | 20 (8.6%) | 95 (40.9%) | **110 (47.4%)** | 7 (3%) |
| Processed meats | 35 (15.1%) | 60 (25.9%) | **113 (48.7%)** | 24 (10.3%) |
| Whole grains | **100 (43.1%)** | 102 (44%) | 21 (9.1%) | 9 (3.9%) |
| Salted foods | 23 (9.9%) | 66 (28.4%) | **131 (56.5%)** | 12 (5.2%) |
| Water | **176 (75.9%)** | 44 (19%) | 9 (3.9%) | 3 (1.3%) |

**Supplementary Table 2.1.2. Response to Nutrition Knowledge Questions for Type of Fats and Recommendation**

| **Type of Fats** | **Recommendation N (%)** | | |
| --- | --- | --- | --- |
|  | **Eat less** | **Don't eat less** | **Not sue** |
| Unsaturated fats | 97 (41.8%) | **97 (41.8%)** | 38 (16.4%) |
| Trans fats | **144 (62.1%)** | 56 (24.1%) | 32 (13.8%) |
| Saturated fats | **133 (57.3%)** | 53 (22.8%) | 46 (19.8%) |

**Supplementary Table 2.1.3. Response to Nutrition Knowledge Questions for Type of Food and Level of Content of Added Sugar**

| **Type of Food** | **Level of Content of Added Sugar N (%)** | | |
| --- | --- | --- | --- |
|  | **High** | **Low** | **Not sure** |
| Low-calorie soft drinks | 166 (71.6%) | **54 (23.3%)** | 12 (5.2%) |
| Yogurt | 32 (13.8%) | **181 (78%)** | 19 (8.2%) |
| Ice cream | **173 (74.6%)** | 41 (17.7%) | 18 (7.8%) |
| Ketchup | **153 (65.9%)** | 56 (24.1%) | 23 (9.9%) |
| Watermelon | 83 (35.8%) | **122 (52.6%)** | 27 (11.6%) |

**Supplementary Table 2.1.4. Response to Nutrition Knowledge Questions for Type of Food and Level of Content of Salt**

| **Type of Food** | **Level of Content of Salt N (%)** | | |
| --- | --- | --- | --- |
|  | **High** | **Low** | **Not sure** |
| Breakfast cereal | **64 (27.6%)** | 128 (55.2%) | 40 (17.2%) |
| Frozen vegetables | 56 (24.1%) | **137 (59.1%)** | 39 (16.8%) |
| Bread | **80 (34.5%)** | 123 (53%) | 29 (12.5%) |
| Baked beans | **87 (37.5%)** | 105 (45.3%) | 40 (17.2%) |
| Red meat | 79 (34.1%) | **122 (52.6%)** | 31 (13.4%) |
| Canned soup | **151 (65.1%)** | 53 (22.8%) | 28 (12.1%) |

**Supplementary Table 2.1.5. Response to Nutrition Knowledge Questions for Type of Food and Level of Content of Fiber**

| **Type of Food** | **Level of Content of Fiber N (%)** | | |
| --- | --- | --- | --- |
|  | **High** | **Low** | **Not sure** |
| Oatmeal | **171 (73.7%)** | 44 (19%) | 17 (7.3%) |
| Bananas | **137 (59.1%)** | 75 (32.3%) | 20 (8.6%) |
| White rice | 68 (29.3%) | **137 (59.1%)** | 27 (11.6%) |
| Eggs | 78 (33.6%) | **122 (52.6%)** | 32 (13.8%) |
| Potatoes with skin | **143 (61.6%)** | 69 (29.7%) | 20 (8.6%) |
| Pasta | 71 (30.6%) | **131 (56.5%)** | 30 (12.9%) |

| **Type of Food** | **Quality as Protein Source N (%)** | | |
| --- | --- | --- | --- |
|  | **Good source** | **Not good source** | **Not sure** |
| Poultry | **195 (84.1%)** | 23 (9.9%) | 14 (6%) |
| Cheese | **120 (51.7%)** | 87 (37.5%) | 25 (10.8%) |
| Fruit | 64 (27.6%) | **130 (56%)** | 38 (16.4%) |
| Baked beans | **103 (44.4%)** | 94 (40.5%) | 35 (15.1%) |
| Butter | 67 (28.9%) | **126 (54.3%)** | 39 (16.8%) |
| Nuts | **130 (56%)** | 69 (29.7%) | 33 (14.2%) |

**Supplementary Table 2.1.6. Response to Nutrition Knowledge Questions for Type of Food and Quality as Protein Source**

**Supplementary Table 2.1.7. Response to Nutrition Knowledge Questions for Type of Food and Being Starchy or Non-Starchy**

| **Type of Food** | **Being Starchy or Non-Starchy N (%)** | | |
| --- | --- | --- | --- |
|  | **Starchy** | **Non-starchy** | **Not sure** |
| Cheese | 45 (19.4%) | **165 (71.1%)** | 22 (9.5%) |
| Pasta | **187 (80.6%)** | 35 (15.1%) | 10 (4.3%) |
| Potatoes | **187 (80.6%)** | 25 (10.8%) | 20 (8.6%) |
| Nuts | 57 (24.6%) | **150 (64.7%)** | 25 (10.8%) |
| Sweet potatoes | **180 (77.6%)** | 30 (12.9%) | 22 (9.5%) |

**Supplementary Table 2.1.8. Response to Nutrition Knowledge Questions for Type of Food and Main Fat Type**

| **Type of Food** | **Main Fat Type N (%)** | | | | |
| --- | --- | --- | --- | --- | --- |
|  | **Polyunsaturated fats** | **Monounsaturated fats** | **Saturated fat** | **Cholesterol** | **Not sure** |
| Olive oil | 98 (42.2%) | **65 (28%)** | 33 (14.2%) | 11 (4.7%) | 25 (10.8%) |
| Butter | 48 (20.7%) | 48 (20.7%) | **79 (34.1%)** | 28 (12.1%) | 29 (12.5%) |
| Sunflower oil | **62 (26.7%)** | 61 (26.3%) | 51 (22%) | 32 (13.8%) | 26 (11.2%) |
| Eggs | 50 (21.6%) | 40 (17.2%) | 37 (15.9%) | **69 (29.7%)** | 36 (15.5%) |

**Supplementary Table 2.1.9. Response to Nutrition Knowledge Statements and Agreement Level**

| **Statement** | **Agreement Level**  **N (%)** | | |
| --- | --- | --- | --- |
|  | **Agree** | **Disagree** | **Not sure** |
| Snack foods or diet foods are always a good choice because they are low in calories | 133 (57.3%) | **59 (25.4%)** | 40 (17.2%) |
| To maintain a healthy body weight, avoid fats at all. | 51 (22%) | **150 (64.7%)** | 31 (13.4%) |
| To maintain a healthy body weight, follow a protein-rich diet. | 131 (56.5%) | **64 (27.6%)** | 37 (15.9%) |
| Eating bread almost always leads to weight gain. | 102 (44%) | **96 (41.4%)** | 34 (14.7%) |
| Eating dietary fiber reduces the likelihood of weight gain. | **140 (60.3%)** | 53 (22.8%) | 39 (16.8%) |

**Supplementary Table 2.2. Nutrition Knowledge Translated into Healthier Behaviors**

| **Behavior** | **Contribution to Weight Maintenance N (%)** | | |
| --- | --- | --- | --- |
|  | **Yes** | **No** | **Not sure** |
| Don't eat while watching TV | **174 (75%)** | 42 (18.1%) | 16 (6.9%) |
| Read food labels | **181 (78%)** | 37 (15.9%) | 14 (6%) |
| Take supplements | 144 (62.1%) | **51 (22%)** | 37 (15.9%) |
| Monitor what you eat | **184 (79.3%)** | 32 (13.8%) | 16 (6.9%) |
| Watch your weight | **174 (75%)** | 43 (18.5%) | 15 (6.5%) |
| Eat throughout the day | 49 (21.1%) | **158 (68.1%)** | 25 (10.8%) |

**Note**: in all the above tables, bolded answers are the correct ones.

**Supplementary Table 3. The Association between Dietary Habits and Stress**

| **Dietary Behavior Question (which Represents a Criterion)** | **Answer (Behavior)** | **Level of Stress N (%)** | | | | **P-value** |
| --- | --- | --- | --- | --- | --- | --- |
|  |  | **Low risk** | **Fragile** | **Challenged** | **High risk** |  |
| Do you eat breakfast? | Yes | 70 (48.6%) | 7 (4.9%) | 13 (9%) | 54 (37.5%) | 0.001 |
|  | Sometimes | 17 (32.1%) | 2 (3.8%) | 4 (7.5%) | 30 (56.6%) |  |
|  | No | 9 (25.7%) | 1 (2.9%) | 3 (8.6%) | 22 (62.9%) |  |
| How many meals do you eat per day? | < 3 | 48 (33.8%) | 7 (4.9%) | 14 (9.9%) | 73 (51.4%) | 0.032 |
|  | 3 | 44 (55%) | 3 (3.8%) | 6 (7.5%) | 27 (33.8%) |  |
|  | > 3 | 4 (40%) | 0 (0%) | 0 (0%) | 6 (60%) |  |
| Do you skip any meals per day? | Yes | 34 (29.8%) | 8 (7%) | 14 (12.3%) | 58 (50.9%) | 0.015 |
|  | Sometimes | 38 (53.5%) | 0 (0%) | 4 (5.6%) | 29 (40.8%) |  |
|  | No | 24 (51.1%) | 2 (4.3%) | 2 (4.3%) | 19 (40.4%) |  |
| Do you eat your main meals during work hours? | Yes, regularily | 26 (51%) | 2 (3.9%) | 2 (3.9%) | 21 (41.2%) | 0.260 |
|  | Sometimes | 40 (40.8%) | 5 (5.1%) | 5 (5.1%) | 48 (49%) |  |
|  | Rarely | 17 (37.8%) | 1 (2.2%) | 6 (13.3%) | 21 (46.7%) |  |
|  | No | 13 (34.2%) | 2 (5.3%) | 7 (18.4%) | 16 (42.1%) |  |
| How often do you eat out? | I don't eat out | 13 (48.1%) | 0 (0%) | 0 (0%) | 14 (51.9%) | 0.122 |
|  | Once a week | 52 (47.7%) | 4 (3.7%) | 9 (8.3%) | 44 (40.4%) |  |
|  | 2-4 times a wee | 29 (33.3%) | 5 (5.7%) | 10 (11.5%) | 43 (49.4%) |  |
|  | Daily | 2 (22.2%) | 1 (11.1%) | 1 (11.1%) | 5 (55.6%) |  |
| Do you spend a lot of time thinking before choosing a food when you're hungry? | Yes | 37 (35.9%) | 5 (4.9%) | 4 (3.9%) | 57 (55.3%) | 0.081 |
|  | Sometimes | 40 (44.4%) | 5 (5.6%) | 11 (12.2%) | 34 (37.8%) |  |
|  | No | 19 (48.7%) | 0 (0%) | 5 (12.8%) | 15 (38.5%) |  |
| Which one do you prefer? | Eating at home | 85 (42.9%) | 9 (4.5%) | 19 (9.6%) | 85 (42.9%) | 0.112 |
|  | Eating from outside | 11 (32.4%) | 1 (2.9%) | 1 (2.9%) | 21 (61.8%) |  |
| Do you read the nutritional labels on the back of products? | Yes | 26 (38.8%) | 2 (3%) | 8 (11.9%) | 31 (46.3%) | 0.506 |
|  | Sometimes | 46 (49.5%) | 4 (4.3%) | 6 (6.5%) | 37 (39.8%) |  |
|  | No | 24 (33.3%) | 4 (5.6%) | 6 (8.3%) | 38 (52.8%) |  |
| How many servings of fruit do you consume per day? | No, I don't eat fruits. | 18 (29%) | 4 (6.5%) | 3 (4.8%) | 37 (59.7%) | 0.042 |
|  | 1-3 | 76 (46.3%) | 6 (3.7%) | 16 (9.8%) | 66 (40.2%) |  |
|  | 4-5 | 2 (33.3%) | 0 (0%) | 1 (16.7%) | 3 (50%) |  |
| How many servings of vegetables do you consume per day? | Never | 6 (23.1%) | 1 (3.8%) | 1 (3.8%) | 18 (69.2%) | 0.017 |
|  | 1 | 3 (42.9%) | 0 (0%) | 1 (14.3%) | 3 (42.9%) |  |
|  | 2-3 | 69 (41.1%) | 8 (4.8%) | 16 (9.5%) | 75 (44.6%) |  |
|  | 4-5 | 18 (62.1%) | 1 (3.4%) | 2 (6.9%) | 8 (27.6%) |  |
|  | > 5 | 0 (0%) | 0 (0%) | 0 (0%) | 2 (100%) |  |
| How many servings of meat and legumes do you consume per day? | Never | 7 (33.3%) | 2 (9.5%) | 0 (0%) | 12 (57.1%) | 0.630* |
|  | 1-3 | 78 (42.4%) | 6 (3.3%) | 14 (7.6%) | 86 (46.7%) |  |
|  | 4-5 | 10 (50%) | 1 (5%) | 5 (25%) | 4 (20%) |  |
|  | > 5 | 1 (14.3%) | 1 (14.3%) | 1 (14.3%) | 4 (57.1%) |  |
| How many servings of dairy do you consume per day? | Never | 15 (34.9%) | 2 (4.7%) | 5 (11.6%) | 21 (48.8%) | 0.136 |
|  | 2-3 | 58 (40%) | 7 (4.8%) | 10 (6.9%) | 70 (48.3%) |  |
|  | 4-5 | 21 (52.5%) | 1 (2.5%) | 5 (12.5%) | 13 (32.5%) |  |
|  | > 5 | 2 (50%) | 0 (0%) | 0 (0%) | 2 (50%) |  |
| How many servings of sugars and sweets do you consume per day? | Never | 16 (44.4%) | 2 (5.6%) | 3 (8.3%) | 15 (41.7%) | 0.293 |
|  | 2-3 | 57 (41.3%) | 8 (5.8%) | 9 (6.5%) | 64 (46.4%) |  |
|  | 4-5 | 21 (43.8%) | 0 (0%) | 7 (14.6%) | 20 (41.7%) |  |
|  | > 5 | 2 (20%) | 0 (0%) | 1 (10%) | 7 (70%) |  |
| How many servings of starches do you consume per day? | Never | 6 (30%) | 2 (10%) | 0 (0%) | 12 (60%) | 0.319 |
|  | 2-3 | 55 (40.4%) | 6 (4.4%) | 10 (7.4%) | 65 (47.8%) |  |
|  | 4-5 | 27 (47.4%) | 2 (3.5%) | 9 (15.8%) | 19 (33.3%) |  |
|  | > 5 | 8 (42.1%) | 0 (0%) | 1 (5.3%) | 10 (52.6%) |  |
| How many glasses of water do you consume per day? | 2-3 | 28 (33.7%) | 1 (1.2%) | 8 (9.6%) | 46 (55.4%) | 0.015 |
|  | 4-6 | 47 (43.1%) | 6 (5.5%) | 11 (10.1%) | 45 (41.3%) |  |
|  | > 6 | 21 (52.5%) | 3 (7.5%) | 1 (2.5%) | 15 (37.5%) |  |

**Supplementary Table 4. The Association between Dietary Habits and Nutrition Knowledge**

| **Dietary Behavior Question (which Represents a Criterion)** | **Answer (Behavior)** | **N** | **Mean** | **SD** | **P-value** |
| --- | --- | --- | --- | --- | --- |
| Do you eat breakfast? | Yes | 144 | 51.68 | 15.87 | 0.175 |
|  | Sometimes | 53 | 52.99 | 18.30 |  |
|  | No | 35 | 55.93 | 16.85 |  |
| How many meals do you eat per day? | < 3 | 142 | 52.81 | 16.27 | 0.501 |
|  | 3 | 80 | 53.16 | 16.87 |  |
|  | > 3 | 10 | 45.53 | 19.08 |  |
| Do you skip any meals per day? | Yes | 114 | 53.35 | 14.86 | 0.674 |
|  | Sometimes | 71 | 51.75 | 18.22 |  |
|  | No | 47 | 52.14 | 18.22 |  |
| Do you eat your main meals during work hours? | Yes, regularly | 51 | 51.51 | 14.54 | 0.037 |
|  | Sometimes | 98 | 49.15 | 16.93 |  |
|  | Rarely | 45 | 58.59 | 15.05 |  |
|  | No | 38 | 55.98 | 18.01 |  |
| How often do you eat out? | I don't eat out | 27 | 50.28 | 14.81 | 0.266 |
|  | Once a week | 109 | 52.23 | 17.25 |  |
|  | 2-4 times a wee | 87 | 53.39 | 16.79 |  |
|  | Daily | 9 | 56.86 | 11.90 |  |
| Do you spend a lot of time thinking before choosing a food when you're hungry? | Yes | 103 | 52.29 | 16.29 | 0.059 |
|  | Sometimes | 90 | 50.59 | 17.63 |  |
|  | No | 39 | 58.16 | 13.83 |  |
| Which one do you prefer? | Eating at home | 198 | 53.46 | 16.96 | 0.062 |
|  | Eating from outside | 34 | 47.72 | 13.46 |  |
| Do you read the nutritional labels on the back of products? | Yes | 67 | 54.91 | 17.00 | 0.039 |
|  | Sometimes | 93 | 53.69 | 17.29 |  |
|  | No | 72 | 49.10 | 14.85 |  |
| How many servings of fruit do you consume per day? | No, I don't eat fruits. | 62 | 48.62 | 15.89 | 0.037 |
|  | 1-3 | 164 | 54.08 | 16.50 |  |
|  | 4-5 | 6 | 53.92 | 22.22 |  |
| How many servings of vegetables do you consume per day? | Never | 26 | 44.43 | 17.81 | 0.050 |
|  | 1 | 7 | 55.46 | 7.23 |  |
|  | 2-3 | 168 | 53.41 | 16.13 |  |
|  | 4-5 | 29 | 54.73 | 18.06 |  |
|  | > 5 | 2 | 51.77 | 26.62 |  |
| How many servings of meat and legumes do you consume per day? | Never | 21 | 39.33 | 13.68 | 0.250* |
|  | 1-3 | 184 | 54.77 | 15.60 |  |
|  | 4-5 | 20 | 47.23 | 20.42 |  |
|  | > 5 | 7 | 51.26 | 19.20 |  |
| How many servings of dairy do you consume per day? | Never | 43 | 50.59 | 18.66 | 0.132* |
|  | 2-3 | 145 | 51.81 | 16.01 |  |
|  | 4-5 | 40 | 58.88 | 14.09 |  |
|  | > 5 | 4 | 41.18 | 25.42 |  |
| How many servings of sugars and sweets do you consume per day? | Never | 36 | 53.01 | 19.52 | 0.310* |
|  | 2-3 | 138 | 50.60 | 16.16 |  |
|  | 4-5 | 48 | 59.58 | 13.54 |  |
|  | > 5 | 10 | 45.65 | 16.62 |  |
| How many servings of starches do you consume per day? | Never | 20 | 38.35 | 17.79 | 0.000 |
|  | 2-3 | 136 | 51.35 | 14.68 |  |
|  | 4-5 | 57 | 59.22 | 17.16 |  |
|  | > 5 | 19 | 56.90 | 16.43 |  |
| How many glasses of water do you consume per day? | 2-3 | 83 | 49.11 | 15.42 | 0.025 |
|  | 4-6 | 109 | 53.96 | 16.52 |  |
|  | > 6 | 40 | 56.24 | 18.18 |  |

* Significance is obtained if regular one-way ANOVA is used

**Supplementary Table 5. The Association between Demographic Variables and Stress**

| **Variable** | **Categories** | **Level of Stress N (%)** | | | | **P-value** |
| --- | --- | --- | --- | --- | --- | --- |
|  |  | **Low risk** | **Fragile** | **Challenged** | **High risk** |  |
| Physical activity level | Low (I rarely exercise) | 25 (35.71%) | 1 (1.43%) | 6 (8.57%) | 38 (54.29%) | 0.315 |
|  | Moderate (I exercise 1-3 times per week) | 63 (45%) | 9 (6.43%) | 10 (7.14%) | 58 (41.43%) |  |
|  | High (I exercise 4 or more times per week) | 8 (36.36%) | 0 (0%) | 4 (18.18%) | 10 (45.45%) |  |
| Number of children | 0 | 23 (33.82%) | 3 (4.41%) | 7 (10.29%) | 35 (51.47%) | 0.051 |
|  | 1-2 | 18 (37.5%) | 3 (6.25%) | 5 (10.42%) | 22 (45.83%) |  |
|  | 3-4 | 30 (44.78%) | 1 (1.49%) | 4 (5.97%) | 32 (47.76%) |  |
|  | 5+ | 25 (51.02%) | 3 (6.12%) | 4 (8.16%) | 17 (34.69%) |  |
| Experience (years) | < 5 | 17 (27.87%) | 3 (4.92%) | 3 (4.92%) | 38 (62.3%) | 0.031 |
|  | 5-10 | 25 (43.86%) | 2 (3.51%) | 6 (10.53%) | 24 (42.11%) |  |
|  | 11-15 | 27 (50.94%) | 3 (5.66%) | 5 (9.43%) | 18 (33.96%) |  |
|  | > 15 | 27 (44.26%) | 2 (3.28%) | 6 (9.84%) | 26 (42.62%) |  |
| Daily work hours | < 6 | 11 (31.43%) | 3 (8.57%) | 0 (0%) | 21 (60%) | 0.203* |
|  | 6-8 | 70 (42.68%) | 7 (4.27%) | 14 (8.54%) | 73 (44.51%) |  |
|  | > 8 | 15 (45.45%) | 0 (0%) | 6 (18.18%) | 12 (36.36%) |  |
| Occupation | Faculty Member | 60 (44.44%) | 5 (3.7%) | 15 (11.11%) | 55 (40.74%) | 0.662 |
|  | Academic Researcher | 4 (36.36%) | 0 (0%) | 1 (9.09%) | 6 (54.55%) |  |
|  | Administrator | 13 (31.71%) | 2 (4.88%) | 2 (4.88%) | 24 (58.54%) |  |
|  | Technician or Administrative Assistant | 14 (48.28%) | 2 (6.9%) | 1 (3.45%) | 12 (41.38%) |  |
|  | Other | 5 (31.25%) | 1 (6.25%) | 1 (6.25%) | 9 (56.25%) |  |

**Supplementary Tables 6. The Association between Other Variables and Nutrition Knowledge**

| **Variable** | **Categories** | **N** | **Mean** | **SD** | **P-value** |
| --- | --- | --- | --- | --- | --- |
| Spending a lot of time thinking before choosing a food (when hungry) | Yes | 103 | 52.29 | 16.29 | 0.059 |
|  | Sometimes | 90 | 50.59 | 17.63 |  |
|  | No | 39 | 58.16 | 13.83 |  |
| Area of KSA | Central | 38 | 53.59 | 14.58 | 0.000 |
|  | Northern | 111 | 48.01 | 15.01 |  |
|  | Southern | 8 | 44.71 | 9.86 |  |
|  | Eastern | 27 | 52.51 | 16.60 |  |
|  | Western | 45 | 64.71 | 17.49 |  |
|  | Outside KSA | 3 | 51.37 | 8.83 |  |
| Opinion regarding the role of nutrition and its impact on health and job performance | Big role | 199 | 53.63 | 16.50 | 0.035 |
|  | Moderate role | 28 | 47.94 | 16.85 |  |
|  | Small role | 5 | 38.35 | 7.28 |  |

| **Regions of Saudi Arabia** | **PA level N (%)** | | | **P-value** |
| --- | --- | --- | --- | --- |
|  | **Low** | **Moderate** | **High** |  |
| Central | 9 (23.7%) | 24 (63.2%) | 5 (13.2%) | 0.874 |
| Northern | 36 (32.4%) | 67 (60.4%) | 8 (7.2%) |  |
| Southern | 2 (25%) | 5 (62.5%) | 1 (12.5%) |  |
| Eastern | 11 (40.7%) | 14 (51.9%) | 2 (7.4%) |  |
| Western | 11 (24.4%) | 28 (62.2%) | 6 (13.3%) |  |
| Outside KSA | 1 (33.3%) | 2 (66.7%) | 0 (0%) |  |

**Supplementary Table 7.1. Otehr Associations: Regions of Saudi Arabia**

| **Daily Work Hours** | **Frequency of Eating Main Meals during Work Hours N (%)** | | | | **P-value** |
| --- | --- | --- | --- | --- | --- |
|  | **Regularly** | **Sometimes** | **Rarely** | **No** |  |
| < 6 | 12 (34.3%) | 13 (37.1%) | 6 (17.1%) | 4 (11.4%) | 0.074 |
| 6-8 | 32 (19.5%) | 73 (44.5%) | 34 (20.7%) | 25 (15.2%) |  |
| > 8 | 7 (21.2%) | 12 (36.4%) | 5 (15.2%) | 9 (27.3%) |  |

**Supplementary Table 7.2. Otehr Associations: Daily Work Hours**
